# A real-time heat strain index using foot temperature and heart rate while wearing personal protective equipment in hot environments

**DOI:** 10.1186/2046-7648-4-S1-A104

**Published:** 2015-09-14

**Authors:** Joo-Young Lee, Siyeon Kim, Joonhee Park, Yutaka Tochihara

**Affiliations:** 1 College of Human Ecology, Seoul National University, Seoul, Republic of Korea; 2 Faculty of Design, Kyushu University, Fukuoka, Japan

## Introduction

Over the past century, a number of indices to assess heat stress and strain in hot environments have been developed, but there are few non-invasive indices to evaluate the heat strain of workers wearing personal protective equipment. ISO 7933 [1] presents an analytical method to determine heat stress using calculation of the predicted heat strain but the calculation is complicated to apply for real-time monitoring. Moran and colleagues [2,3] derived a simple and useful index based on rectal temperature (*T*_re_) and heart rate (HR) (Physiological Strain Index, PSI) but the index is limited at work in fields because of the direct measurement of rectal temperature. The purpose of this study was to present a non-invasive method to monitor heat strain in real-time using foot temperature (*T*_foot_) and HR of workers wearing personal protective equipment with protective boots in hot environments.

## Methods

Three experimental dataset were used in this study. *[Series A] *Eight male students [48.0 ± 16.7 ml.kg^-1^.min^-1 ^in VO_2peak _and 193 ± 8 bpm in HR_max_] participated in 12 experimental conditions: two activities × three clothing levels × two air temperatures (25°C and 32°C with 50%RH). Three types of experimental ensembles were employed: Control (total clothing mass of 590 g except running shoes, 62 % covered of BSA, CBSA [4]), Tyvek condition (787 g, 98 % CBSA), and plastic coverall condition (1,245 g, 98 % CBSA, no evaporation except the face). Two levels of metabolic activities were assigned at 60-min rest and exercise on the treadmill at 6~8 km.h^-1^. *[Series B] *Eight male students different to those in Series A participated in Series B [49.2 ± 6.6 ml.kg^-1^.min^-1 ^in VO_2peak _and 193 ± 7 bpm in HR_max_]. Experimental conditions consisted of an eight conditions: four firefighter's protective equipment conditions (three types of self-contained breathing apparatus (SCBA) conditions were employed with no SCBA condition) × two air temperatures (*T*_a _of 22°C and 32°C with 50%RH). Participants conducted a 30 min exercise on the treadmill at 6 km.h^-1^. *[Series C] *Twelve male firefighters [45.6 ± 7.6 ml.kg^-1^.min^-1 ^in VO_2peak _and 189 ± 7 bpm in HR_max_] participated in two exercise conditions (exercise on the treadmill at 5.5 km.h^-1 ^with/without break) at *T*_a _of 32°C with 43%RH. *T*_re_, eight skin temperatures including *T*_foot_, HR, total sweat rate, subjective perceptions were recorded in all experiments (96 trials in Series A + 64 trials in Series B + 24 trials in Series C = 184 trials). We modified the PSI [2] using *T*_foot _and HR from the first dataset and the second and third dataset were taken to validate the newly modified PSI with original PSI.

## Results

*T*_foot _of 38.0°C and 38.5°C were determined as Alarm and Danger criteria, respectively. The Alarm level was set at the point that *T*_foot _reached *T*_re _during exercise. This level was limited to the conditions of wearing full personal protective equipment (98 % CBSA) at *T*_a _of 32°C. The Danger level was determined at the moments that extreme subjective perceptions (very uncomfortable, very hot, and very hard) were given. The original PSI [2,3] was modified using *T*_foot _and HR, and the modified PSI showed a significant relationship with the original PSI (r = 0.756, *P*<0.05) while exercise wearing PPE at *T*_a _of 32°C.

## Conclusion

The modified PSI using non-invasive variables are valid to predict heat strain for workers wearing full protective equipment including protective boots in hot environments, but cannot be applied to workers wearing light work wear in thermal neutral or cool environments.
